# ‘Pre-optimization’ of the solvent of nanoparticle synthesis for superior catalytic efficiency: a case study with Pd nanocrystals†

**DOI:** 10.1039/d0na01006e

**Published:** 2021-02-17

**Authors:** Lipipuspa Sahoo, Parmeet Kaur Dhindsa, Nihal C. P, Ujjal K. Gautam

**Affiliations:** Department of Chemical Sciences, Indian Institute of Science Education and Research (IISER)-Mohali Sector 81 SAS Nagar Mohali Punjab 140306 India ujjalgautam@gmail.com ujjalgautam@iisermohali.ac.in

## Abstract

In view of a limited rationale available for designing metal nanocrystals (NCs) to achieve high catalytic activities across various chemical transformations, we offer a new perspective on the optimization of the ‘*solvent-of-nanocrystal-synthesis*’ that, to an extent, would help bypass the tedious characterization needs. A systematic improvement in a catalyst is hindered because (i) it relies on size & shape control protocols, surface characterization, understanding molecular transformation mechanisms, and the energetics of the reactant–catalyst interactions, requiring the involvement of different domains experts, and (ii) the insights developed using model reactions may not easily extend to other reactions, although the current studies count on such a hypothesis. In support of (ii), by taking Pd NCs as catalysts and two distinct reaction types, *viz.* Suzuki coupling and nitroarene reduction, we show to what great extent the reaction rates may vary even for the seemingly similar reactions by using the same NCs. More importantly, for challenge (i), we demonstrate how the addition of a single-step to the current protocol of ‘catalyst-synthesis and activity test’ can potentially lead to the development of highly active catalysts by first finding a suitable solvent for the NC synthesis, while such solvent-effects are barely considered unlike the same in organic transformation reactions as a matter of routine, for example.

## Abstract 2

The effect of ‘solvent-of-nanoparticles-synthesis (SONS)’ on their catalytic efficiencies of was studied. The efficiencies widely deviate from what is anticipated, induced by the SONS. An optimization approach to attain high efficiency is proposed.

## Graphical abstract

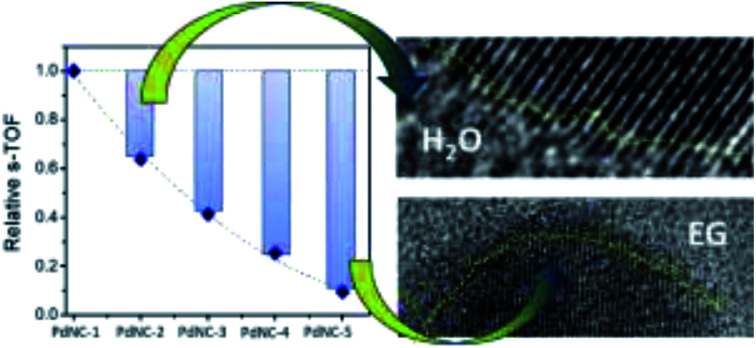

## 1. Introduction

Synthetic methodologies that precisely control the morphological features of the metal nanocrystals (NCs) are important because their catalytic efficiencies are governed by the size, shape, crystallinity, and surface composition.^1–7^ Solution-based strategies have been widely adopted to achieve nanostructures with defined morphologies by adjusting the various synthesis parameters such as precursors, solvents, reducing agents, preparation conditions (*e.g.* temperature, pressure, pH, duration), and stabilizing agents.^8–18^ To head towards a predictable design-driven synthesis of metal nanocatalysts with high catalytic properties, it is necessary to systematically understand the impact of each of these parameters on both the growth of NCs and the subsequent catalytic activities.

There have been studies in the recent past that relate ‘*solvents-of-synthesis*’ to shape and size control of nanocrystals.^19–29^ For example, Douglas *et al.* had explored the relevance of solvent viscosity in the size-controlled synthesis of magnetite NCs.^22^ Likewise, the size of Au NCs has been controlled by varying the composition of the benzene : CHCl_3_ solvent mixture during synthesis.^23^ It has also been shown that the crystallite-size decreases and morphology changes with increasing dipole moment of the solvent.^21,26^ Thus, the solvents, besides the classical controls such as the shape directing agents, can play a critical role in the synthesis of NCs, by adjusting the reactivity of precursors and rate of reactant collision, stabilizing certain facet atoms, *etc.*, and thus altering their nucleation and growth rates. There are, however, far fewer studies that explicitly investigate the influence of ‘*solvents-of-synthesis*’ on the catalytic performance of these NCs.

The synthesis of the NCs and their use in catalysis are two distinct and separate processes that need not have a simple correlation. This is because while size and shape are somewhat macroscopic manifestations, there are many manipulations at a ‘few-atom-level’ on the catalyst NCs that the ‘*solvents-of-synthesis*’ may bring about, which are difficult to be envisioned. The solvent can help create defects, occlusions, and cavities on the nanocrystal surface to potentially alter the catalytically active sites.^30,31^ As an example, remarkable changes in the defect concentration and surface properties of ZnO nanostructures were observed to influence the catalytic properties from the variation of the ‘*solvent-of-synthesis*’ due to defect engineering.^31^ Similarly, ‘*solvent-of-synthesis*’ could generate structural defects related to sulphur vacancies during the synthesis of CdS nanoparticles, and Rh nanocrystals with different shapes are obtained in different solvents, although such studies on metal nanocrystals are limited.^29,30^ In addition, some solvents have a stronger affinity for certain surface atoms to remain there even after removal from the mother liquid, just like the capping agents, to considerably modulate the electron density around it, consequently altering the activation barrier for the catalytic reaction.^19,32,33^ Finally, the ‘*solvents-of-synthesis*’ also influence the stability and coverage density of the capping agents around the NCs.^3,20^ This can severely impart catalysis since the eventual catalytic capability of NCs is greatly dependent on the thickness of the shell.^32,33^ The solvents also affect the orientation of the capping agents by virtue of their polarity.^34^

It is therefore evident that solvents operate not only towards achieving desired nanoparticle morphologies but also as a key to modulate the catalytic properties. If this be the case, making a suitable choice of solvent during NC synthesis becomes more important for attaining desired catalytic activity, and yet, the optimization of the ‘*solvent-of-synthesis*’ is not a general practice. In contrast, it is routine to optimize the solvents to be used for organic transformations catalysed by the NCs to come up with the most effective conditions, as was recently reviewed, for instance, illustrating the role of solvents in chemical transformations.^35^ The catalytic reaction rates and yields are susceptible to solvent effects.^36,37^ Naturally, a question arises as to whether the optimization of the ‘*solvent-of-synthesis*’ should be exercised as an essential and integral step for attaining the desired activity of the NCs, and if so, to what extent would it improve catalytic efficiency.

In this context, we present a systematic study that offers important insights into the role of ‘*solvent-of-synthesis*’ towards imparting high catalytic efficiency on Pd NCs. Herein, quasi-spherical palladium nanocrystals were prepared by reducing a palladium salt in mixtures of two common solvents, water and ethylene glycol (EG) with significantly different viscosities. The systematic increase in the fraction of EG in the mixture of H_2_O–EG led to a corresponding increase of the Pd NC average diameter, as anticipated. However, importantly and in a rather unpredictable manner, using two key Pd catalysed reactions, Suzuki–Miyaura cross-coupling and the reduction of nitroarene compounds, we show that the changes in the catalytic properties of the Pd NCs prepared in different solvent mixtures are way more different than what can be anticipated from the consideration of their sizes alone. In other words, despite having the same preparation conditions but for the *solvent-of-synthesis*, the catalytic activity per surface Pd-atom is dramatically different and varies monotonously over 10 times from 0.1 to 1.0 in a relative scale for Suzuki coupling. In a similar way for nitroarene reduction, the activity may vary from 0.2 to 1.0, but then not monotonously. The deviations from the expected values have been ascribed to changes in the (a) nature and (b) oxidation state of the surface atoms, (c) PVP–Pd interactions, and (d) deviations from the spherical shape at an atomic scale which originated during synthesis. Since such aspects are not predictable at the current state of research, the findings highlight how optimizing an appropriate solvent for the synthesis of NCs can potentially improve their catalytic efficiencies and the screening of the ‘*solvents-of-synthesis*’ ought to be an integral part of an investigation, just as the screening of ‘*solvents-of-reaction*’ in the conventional organic transformations.

## 2. Experimental section

### 2.1 Chemicals

Palladium-(II) chloride (PdCl_2_, 99.99%), phenylboronic acid (C_6_H_7_BO_2_, >98%), and 4-iodotoluene (C_7_H_7_I, 98%) were purchased from Alfa Aesar. Polyvinylpyrrolidone (PVP, *M*_W_ = 40 000), 4-nitrophenol (O_2_NC_6_H_4_OH, ≥99%), substituted nitroarenes, and sodium borohydride (NaBH_4_, >99%) were purchased from Sigma Aldrich. Ethanol (ACS grade), ethylene glycol (ACS grade), and isopropanol (ACS grade) were purchased from Merck. Ethyl acetate (LR) and hexane (LR) were purchased from Rankem. Anhydrous potassium carbonate (K_2_CO_3_, 99%) was purchased from Loba Chemie. Hydrochloric acid (HCl, 35% AR) was purchased from Himedia. Ultrapure water with a resistivity of 18.2 MΩ cm^−1^ was used for all experiments. All chemicals were used without further purification.

#### 2.1.1 Synthesis of PdNCs

Solvothermal synthesis of PdNCs was performed in solvent mixtures of water containing ethylene glycol (EG) in varying percentages of 0%, 25%, 50%, 75%, and 100%. In a typical synthesis, 2 g PVP (*M*_W_ = 40 000) was dissolved in 20 mL solvent by magnetic stirring at 700 rpm until a clear solution was obtained. PdCl_2_ (45 mg) was dissolved in concentrated HCl (11.6 M, 250 μL) by ultrasonication for 5 minutes to convert it into H_2_PdCl_4_. 10 mL of the solvent mixture was added to H_2_PdCl_4_ and stirred for 10 minutes. This H_2_PdCl_4_ solution was slowly added dropwise to the PVP solution over a period of 3 minutes under magnetic stirring and stirring was continued for another 20 minutes to ensure homogeneity. The resulting brown solution was transferred into a 48 mL Teflon-lined stainless-steel autoclave and kept inside a preheated oven at 200 °C for 12 hours. The obtained black solution was centrifuged at 13 500 rpm for 30 minutes to separate PdNCs and washed 5 times with water and 2 times with ethanol to remove excess PVP. Finally, the PdNCs were redispersed in 2 mL ethanol and stored for further use. The Pd NCs were labeled according to the ‘solvent-of-synthesis’ as PdNC-1 for 0% EG, PdNC-2 for 25% EG, PdNC-3 for 50% EG, PdNC-4 for 75% EG, and PdNC-5 for 100% EG present in the solvent mixture.

#### 2.1.2 Materials characterization

Transmission electron microscope (TEM) and high-resolution TEM (HRTEM) images were taken using a JEOL F200 TEM operating at 200 kV. Powder X-ray diffraction patterns were recorded with a Rigaku Ultima IV diffractometer with Cu Kα X-ray radiation (generator power setting: 40 mA and 40 kV) at a scan rate of 2° min^−1^. Infrared spectra were recorded between 700 and 4000 cm^−1^ using a Perkin Elmer FT-IR spectrometer with samples prepared as KBr pellets. UV-vis absorption spectra were collected using a Perkin Elmer spectrophotometer with a quartz cuvette of path length 10 mm. ^1^H NMR spectra were collected with a 400 MHz Bruker Biospin Advance III FT-NMR spectrometer. NMR shifts have been presented as delta (*δ*) units in parts per million (ppm). X-ray photoelectron spectroscopy measurements were performed using an AXIS ULTRA XPS spectrometer. At first, a Shirley background was subtracted from the XPS data and then, the XPS curves were deconvoluted using Voigt function.

#### 2.1.3 Catalytic reduction of nitroarenes

The PdNC dispersion in water (0.4 mg mL^−1^) was prepared by ultrasonication. In a standard quartz cuvette of path length 10 mm, 2.7 mL 4-nitrophenol (0.1 mM) solution was mixed with 0.3 mL NaBH_4_ (0.3 M) solution at room temperature (*T* = 26 ± 1 °C). To this, 10 μL of Pd NC dispersion (0.4 mg mL^−1^) was added. The cuvette was placed inside a UV-vis spectrophotometer and the reaction was monitored by recording the changes in the absorption spectra with time. As the reaction progressed, the bright yellow solution gradually became colourless. The apparent rate constant, *k*, was calculated by following the first-order rate law.^38^
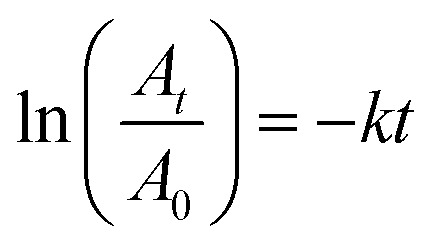
where *A*_0_ and *A*_*t*_ are absorbance values (at 400 nm) representing the reactant concentrations in the beginning and at time *t* respectively. Similarly other nitroarenes were tested with the following reactants and catalyst concentrations (0.5 mM 3-nitrophenol + 0.3 M NaBH_4_ + 9 μL Pd; 0.5 mM 2-nitrophenol + 0.3 M NaBH_4_ + 9 μL Pd; 0.1 mM 2-hydroxy-5-benzaldehyde + 0.3 M NaBH_4_ + 10 μL Pd; and 0.2 mM 4-nitroaniline + 0.3 M NaBH_4_ + 6 μL Pd). Note that the catalyst concentration for a particular substrate was kept constant for meaningful comparison. However, for different substrates having different reactivities, concentrations were varied as otherwise few reactions complete within seconds and a few others went for several hours.

#### 2.1.4 Suzuki–Miyaura cross-coupling reactions

In a typical reaction, 4-iodotoluene (1 mmol), phenylboronic acid (1.2 mmol), and K_2_CO_3_ (2 mmol) were taken in a round-bottomed flask (10 mL) followed by the addition of 2 mL ethanol and 2 mL water. 1 mg of Pd NC dispersion in 100 μL ethanol was added to the reaction mixture and stirred at 700 rpm at room temperature (*T* = 26 ± 1 °C). The progress of the reaction was monitored using the thin-layer chromatography technique. The reaction was allowed to proceed for the desired amount of time and the product was collected by evaporation of ethanol using a rotary evaporator and extracted in ethyl acetate. The purified product was separated using column chromatography. The product so obtained was dried under high vacuum and analysed by NMR spectroscopy using CDCl_3_ as a solvent.

## 3. Results and discussion

### 3.1 Characterization of the PdNCs

In order to understand the influence of the solvent used in nanocrystals synthesis on their catalytic performances, several sets of Pd NCs were prepared using the same precursors and stabilizing agents but systematically varying the solvent used for synthesis. The Pd NCs were obtained by a hydrothermal method, where the amounts of Pd precursor and PVP acting as the stabilizer cum reducing agent were held constant, while the solvent composition was varied by using water and ethylene glycol (H_2_O : EG) mixture in a different ratio (volume/volume).

**Fig. 1:**
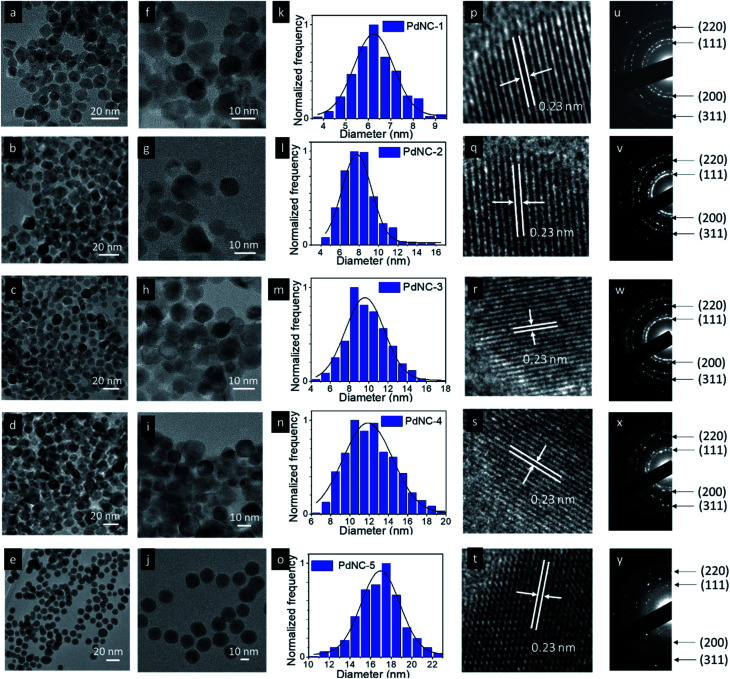
Low magnification TEM images of (a and f) PdNC-1, (b and g), PdNC-2, (c and h) PdNC-3, (d and i) PdNC-4, (e and j) PdNC-5. Particle size distribution histogram of (k) PdNC-1, (l), PdNC-2, (m) PdNC-3, (n) PdNC-4, (o) PdNC-5. HRTEM images of (p) PdNC-1, (q), PdNC-2, (r) PdNC-3, (s) PdNC-4, (t) PdNC-5 and indexed SAED patterns of (u) PdNC-1, (v) PdNC-2, (w) PdNC-3, (x) PdNC-4, (y) PdNC-5.

The TEM images of the PdNCs prepared by varying the H_2_O : EG ratio (from 1 : 0 to 0 : 1) are given in Fig. 1. The low magnification images show that all the NCs have an average spherical shape with a nearly uniform size distribution (Fig. 1a–j and S1–S5†). The corresponding particle size distribution histograms provide clear evidence of the dependence of the size of Pd NCs synthesized by varying the H_2_O : EG ratio (Fig. 1k–o). The sizes of the PdNCs were found to be dependent on the composition of the solvent used in synthesis. Increasing the viscosity of the EG–water mixture led to the formation of bigger nanocrystals. The average particle size gradually increased with increasing fraction of EG in the solvent mixture from 0% (6.4 ± 1.0 nm, PdNC-1), 25% for (7.9 ± 1.6 nm, PdNC-2), 50% (9.8 ± 2.1 nm, PdNC-3), and 75% (12.4 ± 2.6 nm, PdNC-5) to 100% (16.9 ± 2.2 nm PdNC-5). These can be attributed to the slower nucleation rate in highly viscous, high-boiling solvents, which limits the collision of reactive species to form a smaller number of nuclei formation, leaving more precursor for growth and yielding larger nanocrystals.^39,40^

**Fig. 2:**
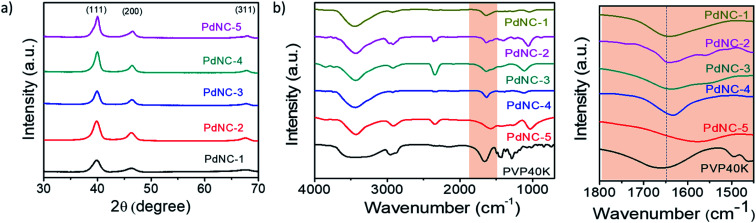
(a) PXRD patterns of the different PdNCs. (b) FT-IR spectra of pure PVP and PdNCs. (c) The highlighted –C=O bonding region in (b).

The high-resolution TEM images recorded on single particles revealed the presence of clear lattice fringes with a spacing of 0.23 nm which can be ascribed to the (111) planes of face centered cubic (FCC) Pd (Fig. 1p–t). The selected area electron diffraction (SAED) patterns, acquired on these samples, displaying diffuse rings can be indexed using the diffraction planes of FCC-Pd (Fig. 1u–y). Powder X-ray diffraction (PXRD) patterns were further recorded to confirm the phase purity of the samples. The peaks near 39.9°, 46.4°, 67.9° can be assigned respectively to the (111), (200), (220) planes of FCC-Pd (JCPDS number-88-2335, Fig. 2a). The relatively sharp peaks with increasing EG concentration indicate that the crystallite size gets bigger from PdNC-1 to PdNC-5.

The Pd–PVP interactions were investigated using FT-IR spectroscopy. Fig. 2b shows a series of FT-IR spectra of the PdNCs and pure PVP recorded between 700 and 4000 cm^−1^. For pure PVP, the absorption band located around 1660 cm^−1^, 1286 cm^−1^, 2953 cm^−1^ can be ascribed to the stretching vibration of the –C=O, –C–N, and –C–H in the pyrrolidone group.^41^ The presence of these signature peaks in all the PdNCs confirms the presence of PVP coating on the surface of Pd even after several washing steps by water and ethanol, forming an organic shell irrespective of the composition of the solvent used during Pd NCs synthesis. The Pd NCs show clear –C=O stretching bands at around 1641, 1641, 1637, 1633, 1577 cm^−1^ respectively for samples PdNC-1 to PdNC-5. The pronounced red-shifts of the C=O stretching frequencies for the PdNCs as compared to pure PVP confirm a strong binding of the PVP on the surface of Pd NCs *via* the pyrrolidone –C=O lone pair in oxygen, which decreases the electron density in the carbonyl bond and thus the vibration energy.^41^ The –CO–Pd interactions are however not uniform in the samples, as evident from (i) the different extents of red-shifts in the 19–83 cm^−1^ range and (b) the splitting of the –C=O stretching peaks. The partial donation of oxygen lone pair to Pd causes the –C=O peak to split into two peaks, as prominent in PdNC-2 and PdNC-3 centered at around 1633 cm^−1^ and 1577 cm^−1^, although the intensity ratio varies depending on the interaction.^42,43^

### 3.2 Catalytic performances of the PdNCs

We examined the catalytic behaviour of the PdNCs during borohydride reduction of nitroarenes and Suzuki–Miyura cross-coupling reactions to understand if their performances are governed by their sizes alone or there are additional effects imparted by the ‘*solvents-of-synthesis*’ and if so, to what extent.

#### 3.2.1 Reduction of nitroarenes

**Fig. 3:**
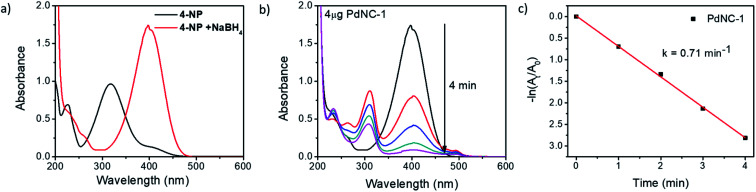
(a) UV-vis absorption spectra of 4-NP before and after the addition of NaBH_4_, (b) time-dependent UV-vis absorption spectra of the 4-NP in presence of NaBH_4_ and 4 μg of PdNC-1 and (c) the corresponding rate-constant plot of −ln(*A*_*t*_/*A*_0_) *vs.* time.

The nitroarene reduction reaction, besides being a widely used model reaction, can be used with suitable modifications to obtain industrially important chemicals.^44–46^ Although the reduction of 4-NP is feasible thermodynamically even in absence of a catalyst due to the favourable reduction potential of the reactants (H_3_BO_3_/BH_4_^−^ = −1.33 V and 4NP/4AP = 0.76 V *versus* NHE), the reaction kinetics is extremely slow, necessitating catalytic assistance. The reaction is usually carried out in an aqueous medium under ambient conditions, and its kinetics can be monitored by recording the temporal UV-vis absorption spectrum. The UV-vis spectrum of 4-NP shows an absorption peak at ∼317 nm, which shifts to ∼400 nm in presence of NaBH_4_ due to the formation of the 4-nitrophenolate ion (Fig. 3a).

**Fig. 4:**
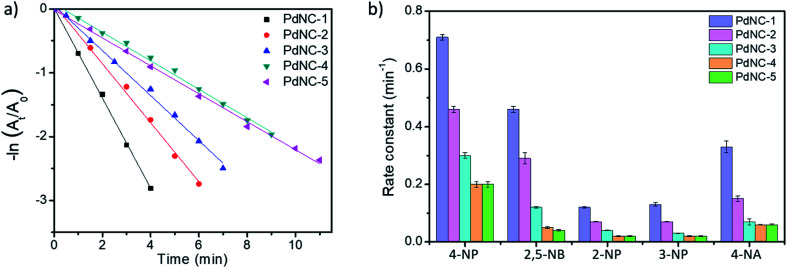
(a) Comparative rate constant plot of −ln (*A*_*t*_/*A*_0_) *vs.* time for 4-NP reduction using the different PdNCs, (b) comparison of the rate-constants exhibited by the different PdNCs for the reduction of 4-NP, 2,5-NB, 2-NP, 3-NP and 4-NA.

To compare the catalytic activities, 4 μg of various Pd NCs and a large excess of NaBH_4_ were used in all the reactions to maintain a pseudo first-order kinetics with respect to the 4-NP concentration. Using PdNC-1, as shown in Fig. 3b, the intensity of the 400 nm peak gradually decreased with the progress of the reaction and a peak corresponding to 4-aminophenol (4-AP) emerged at ∼305 nm.^47^ The reduction completed within 240 seconds, and considering 1st order kinetics, the rate constant was calculated to be 0.71 min^−1^ (Fig. 3c). The data for the reaction using PdNC-2, PdNC-3, PdNC-4, and PdNC-5 are given in Fig. S6† and the corresponding rate constant plots are compared in Fig. 4a. As can be seen (also from Table S1†), the rate constant values are dependent on the ‘solvent-of-synthesis’ for the PdNC synthesis and decrease from 0.71 min^−1^ (for PdNC-1) monotonously to 0.2 min^−1^ (for PdNC-5) by increasing the ethylene glycol percentage in the solvent mixture.

In the same fashion, we have further examined the reduction of other nitroarenes such as 2-hydroxy-5-nitrobenzaldehyde (2,5-NB), 2-nitrophenol (2-NP), 3-nitrophenol (3-NP), and 4-nitroaniline (4-NA) using these PdNCs (Fig. S7–S10†). The corresponding rate constants for 4-NP, 2,5-NBA, 2-NP, 3-NP, and 4-NA are compared in Fig. 4b and in Table S1.† It was observed that the rate constant values follow the same order of PdNC-1 > PdNC-2 > PdNC-3 > PdNC-4 > PdNC-5 for all five of them.

#### 3.2.2 Suzuki–Miyaura cross-coupling reaction

To further inspect the generality of the catalytic behaviour of PdNCs, Suzuki–Miyaura cross-coupling reactions were performed. The easily available reagents, mild reaction conditions, and the ability to tolerate a wide range of functional groups are few of the key advantages in employing the Suzuki–Miyaura coupling reaction to obtain pharmaceutically important compounds.^48–50^ Therefore, in order to compare the catalytic efficiencies of the PdNCs, Suzuki–Miyaura coupling reactions were performed with 4-iodotoluene (1 mmol) and phenylboronic acid (1.2 mmol), in presence of K_2_CO_3_ (2 mmol) as a base at room temperature. Due to the increasing importance of green solvents in organic synthesis, the coupling reactions were carried out in benign aqueous-based water–ethanol (1 : 1) solvent mixtures, thus avoiding the traditionally used organic solvents, such as toluene or THF.^50,51^ Furthermore, for convenience in comparison, all reactions were performed using 0.8 mol% PdNCs with respect to 4-iodotoluene. The reaction was quite favourable using PdNC-1 with a yield of 95% within 25 min. However, the reaction became progressively slower in the order PdNC-1 > PdNC-2 > PdNC-3 > PdNC-4 > PdNC-5 as the ethylene glycol content increased in the ‘*solvent-of-synthesis*’. The efficiency was evaluated by computing turnover frequency (TOF), where TOF is defined as the moles of product formed per moles of catalyst atoms per hour as given below:^52^
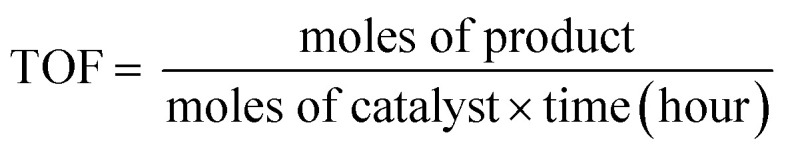
where moles of the product was calculated as yield times the moles of 4-iodotoluene taken. The TOF values for the different PdNCs are compared in Table 1. The TOF for PdNC-1 was the highest (243 h^−1^), which is 1.8 (128 h^−1^), 3.6 (67 h^−1^), 7.1 (34 h^−1^) and 27 (9 h^−1^) times higher than that of PdNC-2, PdNC-3, PdNC-4 and PdNC-5 respectively. The PdNC-5 sample prepared in pure ethylene glycol solvent exhibited the lowest activity with only 65% yield even after 7 h of reaction time.

**Table 1:** Suzuki–Miyaura cross-coupling reaction using 0.1 mg of PdNCs

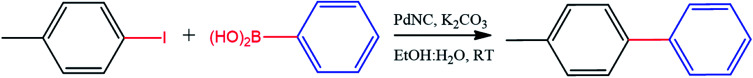				
Catalyst	Time (min)	Conversion (%)	Yield (%)	TOF (h^−1^)
PdNC-1	25	<99	95	243
PdNC-2	45	<99	90	128
PdNC-3	90	<99	95	67
PdNC-4	180	<99	95	34
PdNC-5	420	<70	65	9

### 3.3 Size-normalized catalytic activity of the PdNCs and digression from expectancy

The above findings demonstrate that the Pd nanocrystals obtained using water as the ‘*solvent-of-synthesis*’ exhibit the highest catalytic efficiency for both the Suzuki–Miyaura coupling and nitroarene reduction reactions as compared to the ones prepared in either pure ethylene glycol or mixtures of ethylene glycol and water. Although such a trend is expected as the sizes of nanocrystals increases from PdNC-1 (∼6.4 ± 1.0 nm) to PdNC-5 (∼16.9 ± 2.2 nm) offering a lesser number of surface atoms for participation in the reactions, it is still not clear whether the descending activity is related to their sizes alone or whether there are other influencing factors also, and if so, to what extent such effects can dominate. Therefore, we have normalized the respective TOFs with the fraction of the surface Pd atoms (s-TOF, see details in ESI note-1 and Table S2†) with the assumption that only these atoms would participate in catalysis, and if the size is the principal factor, the s-TOF values should emerge as nearly same for all PdNCs for a single reaction. The number of surface atoms were calculated by assuming a round shape for the PdNCs so that any deviation from sphericality may also be attributed to the effect of the ‘*solvent-of-synthesis*’.

**Fig. 5:**
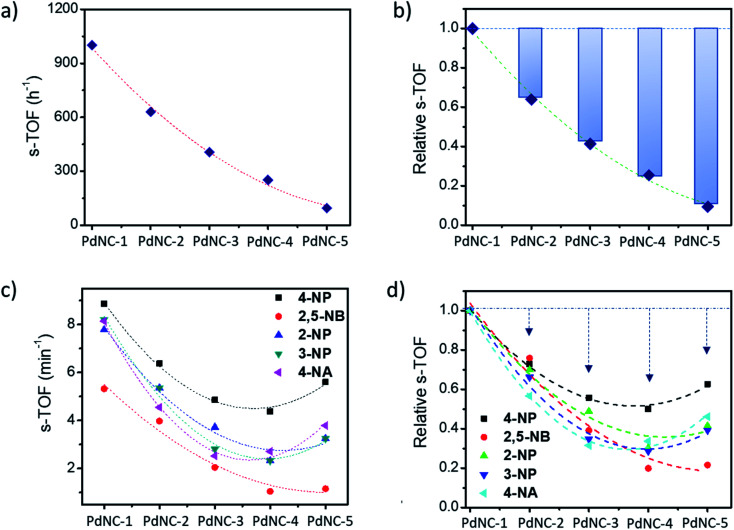
(a) Surface atom normalized TOF (s-TOF) of the various PdNCs for the Suzuki–Miyaura cross-coupling reaction, (b) corresponding relative s-TOF values with respect to the s-TOF value of PdNC-1 for showing the deviation from the expected values, (c) s-TOF values of PdNCs for the nitroarene reduction reaction, and (d) corresponding relative s-TOF values with respect to the s-TOF value of PdNC-1.

Fig. 5a and b, respectively, show the absolute s-TOFs and the relative s-TOF values with respect to the most efficient PdNC-1 obtained using pure water as the ‘*solvent-of-synthesis*’ for Suzuki coupling reactions. The s-TOF value for PdNC-1 was estimated to be 1012 h^−1^ which is 1.6, 2.4, 3.9, 10.4 times higher than that of the PdNC-2, PdNC-3, PdNC-4, and PdNC-5 respectively (Table S3†). This trend however is rather different in the case of nitroarene reduction reactions even though there is a nearly 5–10 fold decrease in s-TOF values (Fig. 5c and d). Here too, the PdNC-1 exhibits the highest s-TOF values for the different reactions such as 8.9 min^−1^, 5.3 min^−1^, 7.8 min^−1^, 8.2 min^−1^, and 8.1 min^−1^ for 4-NP, 2,5-NBA, 2-NP, 3-NP, and 4-NA reduction respectively (Table S4 and S5†). Still, unlike the Suzuki coupling reaction, the s-TOFs for other PdNCs follow a peculiar pattern by decreasing first and increasing at the end in the order of PdNC-1 > PdNC-2 > PdNC-3> PdNC-4 < PdNC-5. The increased s-TOF with PdNC-5 is more prominent for 4-NP and 4-NA reduction. Furthermore, the relative variation in s-TOFs is different while using different reactants and PdNCs as seen from the relative s-TOF values in Fig. 5b and Table S6.† For example, even though the plots for 4-NP and 4-NA appear similar, the maximal s-TOF variation in 4-NP is ∼40% and in 4-NA is ∼60%.

Overall, the s-TOF analysis for Suzuki coupling and nitroarene reduction reactions brings forth the following aspects: (i) the reactivity of the surface atoms in the PdNCs obtained using different ‘*solvents-of-synthesis*’ is different and (ii) the surface reactivity is reactant specific and need not follow the same trend for different reactions. (iii) Furthermore, observation (i) is credible even though it is based on crude estimations of surface atoms because any error in estimations responsible for the observed trend would otherwise have led to the same trends in the reactivity patterns of the PdNCs for both Suzuki coupling and nitroarene reduction, which is clearly not the case (Fig. 5b and d).

### 3.4 Origin of differential catalytic activity and the effect of solvent ‘pre-optimization’

Since the s-TOF analysis clearly demonstrates that there are other factors than the size of the PdNCs alone which influences their reactivity in a remarkable way, questions arise as to what might have led to such effects. From further analysis of the samples, we suggest the following primary factors that broadly influence a deviation from the expected behaviour.

#### 3.4.1 PVP binding to the nanocrystals

**Fig. 6:**
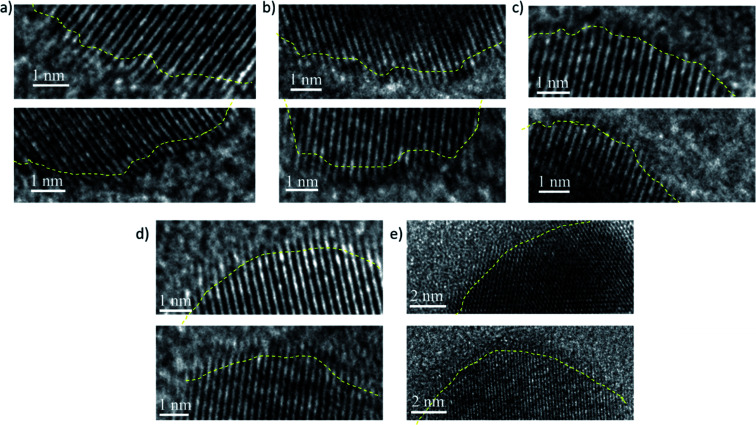
Typical high-resolution TEM images recorded at the nanocrystal edges showing the presence of defects and the different extent of deviations from an ideal spherical shape for (a) PdNC-1, (b) PdNC-2, (c) PdNC-3, (d) PdNC-4 and (e) PdNC-5. The yellow lines a guide to the eye.

**Fig. 7:**
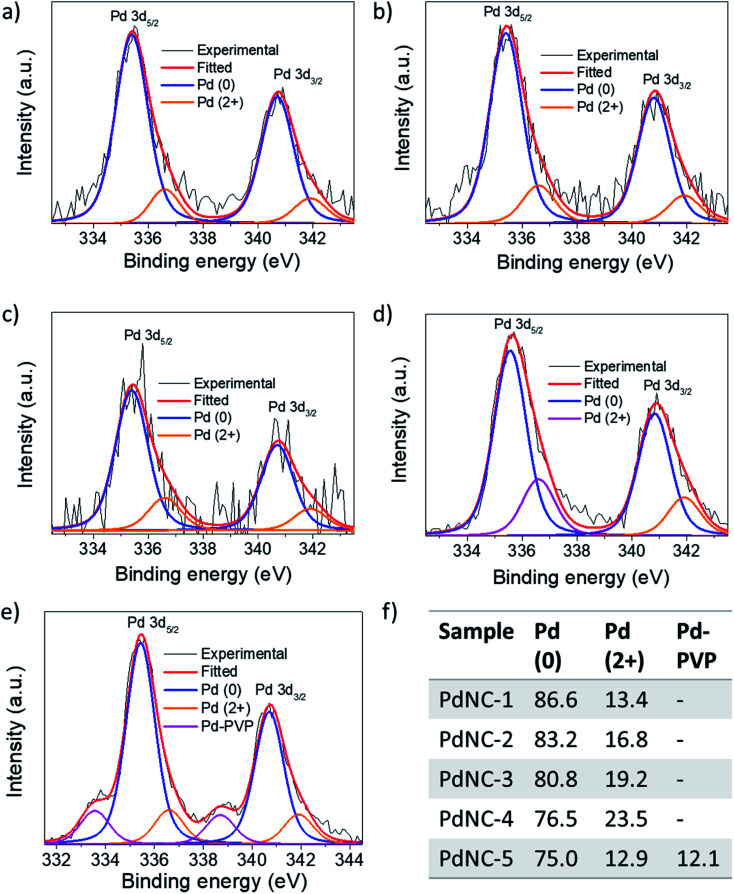
High-resolution XPS spectra in the Pd 3d region of (a) PdNC-1, (b) PdNC-2, (c) PdNC-3, (d) PdNC-4, and (e) PdNC-5 showing the presence of Pd (0) as well as Pd (2+) species. (f) The corresponding percentage of the abundance of each species calculated from Pd 3d_5/2_ peaks.

The stabilizers prevent the facile diffusion of the reactant molecules towards the catalyst surface, thereby inhibiting their performance. Even though the conditions for the synthesis of the PdNCs are identical except for the ‘*solvent-of-synthesis*’, PVP is expected to exhibit different capping ability in different solvents. For example, the PVP–PVP interactions are much stronger in water, which makes the ligand layer on the NCs rigid and restricts the growth of particles leading to the formation of small size particles. On increasing the fraction of EG, interactions between PVP ligands capped on PdNCs become weaker yielding larger particles. We have examined the PVP–Pd interactions by using FT-IR spectroscopy, as previously described in Fig. 2b. Therein, it was observed that the –CO–Pd interactions are not uniform in the samples, as evidenced by the different extent of red-shifts of the C–O stretching peaks (in the 19–83 cm^−1^ range) and the splitting of these peaks. The partial donation of the oxygen lone pair of PVP to Pd has caused the –C=O peak to split into two. The effect is particularly prominent in PdNC-2 and PdNC-3 where the split peaks are centered at 1633 cm^−1^ and 1577 cm^−1^ respectively, although the ratio of their intensities exhibited variation depending on the interaction. Now depending on how the PVP shell orients on the PdNC surface, we have observed the following differences with a strong potential bearing on catalytic properties:

(i) Deviation from spherical shape: during the growth of the PdNCs, if the PVP binding is not uniform and similar for all PdNCs at the intermediate stages, the particles will tend to grow further and more easily in those places on the surface where the binding is weak. Furthermore, in these places, there will be the presence of solvent molecules, water, or the EG–water mixture in different ratios based on the ‘*solvent-of-synthesis*’, which in turn will affect the growth rate of the surface. Thus, there is a strong possibility that the particles may grow out of the spherical shape if looked at microscopically near the surface. As can be seen from Fig. 6 and S11–15,† the surface of the PdNC-1 consists of abundant defects including edges and corner atoms with a low coordination number. On the other hand, in the case of PdNC-5, the surface was observed to be rather smooth. The extent of surface roughness is different for all the PdNCs. Thus, the different extent of deviation from a spherical shape can also be considered as an influence of the ‘*solvent-of-synthesis*’, commensurating with the goal of elucidating the solvent effect. The deviation indicates that the composition of the surface atoms, such as the ratio of the planner atoms, edge atoms, corner atoms *etc.*, in these PdNCs is different. Since catalytic properties are strongly dependent on the nature of surface atoms, the catalyst particles obtained from different ‘*solvents-of-synthesis*’ are also vastly different despite using the same precursors and the capping agents.

(ii) The noble metal surfaces are usually oxidized to some extent due to the presence of otherwise unsaturated dangling bonds.^53^ As stated in (i), if the composition of the surface atoms is different, it is also expected that the surface will oxidize to a different extent. We have carried out XPS measurements to analyze the chemical nature and the electronic properties of the PdNCs. The core-level high-resolution XPS spectra of the Pd 3d region for all PdNCs are given in Fig. 7. The asymmetric peak-broadening towards higher binding energy primarily indicates the presence of higher Pd oxidation states in all samples. The deconvolution of both Pd 3d_5/2_ and Pd 3d_3/2_ peaks shows the presence of two different peaks for samples PdNC-1 to PdNC-4 (Fig. 7a–d). The peaks near ∼335.4 and ∼340.7 eV are assigned to the metallic Pd (0) species.^47,54^ The peaks near 336.6 and 341.9 eV are due to the presence of Pd (2+) species formed because of partial surface oxidation.^47,54^ Interestingly, deconvolution of the Pd 3d peak of PdNC-5 shows that along with Pd (0) and Pd (2+) peaks, two new peaks near 333.5 and 338.7 eV are present (Fig. 7e). These peaks have possibly arisen due to a strong interaction between Pd and PVP, where electron density from the lone pair of oxygen in PVP has been partially transferred to Pd. Such a peak is not clearly visible in other samples and has not been reported so far, to the best of our knowledge. However, the transfer of oxygen electrons to Pd has been observed in the literature from the shift in the oxygen 1s XPS peak.^42,55^ The XPS results agree well with the FT-IR results as the –CO peak is shifted to the lowest energy in the case of PdNC-5. The overall major features from the XPS analysis are that with increasing EG percentage in the ‘*solvent-of-synthesis*’ from PdNC-1 to PdNC-4, the asymmetry in the Pd peaks increases, which suggests a different extent of surface oxidation in them. On integrating the area under the peaks, it was observed that the Pd (2+) percentage increases from 13.4% to 23.3% for PdNC-1 to PdNC-4 as shown in Fig. 7f. However in the case of PdNC-5, along with 12.9% surface oxidised Pd, another 12.1% contribution comes from Pd–PVP interactions.

## 4. Conclusions and outlook

In conclusion, we have observed that the ‘*solvent-of-synthesis*’ for noble metal nanocrystals plays a significant role in improving their catalytic properties. In different ‘*solvents-of-synthesis*’, surfaces of the Pd NCs become catalytically distinct and can enhance their activity by up to 10 times per unit surface area (in addition to enhancements due to size-miniaturization and surface area increase), even though the exact same conditions and reagents were employed for their synthesis. Furthermore, we have shown that the nature of such ‘*catalytic-enhancement*’ is profoundly dependent on the reactants and cannot be stereotyped even for similar reaction types. We have attributed the catalytic enhancements to the tailoring of the intra-capping-agent interactions that depend on the ‘*solvents-of-synthesis*’ and goes on to influence its ability to cap the nanocrystal. As a result, we have observed different extents of oxidation of the surface atoms and microscopic deviations from an apparent *spherical-shape* of the NCs, suggesting a possibility to engineer the surface defects.

In order to examine the nature of the nanocrystal surfaces which affects their catalytic activities such as the catalyst-capping agent interactions, surface atom types, surface vacancies, nature of the oxidation states, coordination number of the active sites *etc.*, a number of advanced analysis techniques and relevant expertise are essential. The undertaking of such experiments on a regular basis is not easy for most researchers, since the same has to be carried out for each set of catalyst NCs and (ii) even if it is done, it may not be relevant for another catalytic reaction, as we have shown here. Therefore one convenient way to avoid this exhaustive process to achieve high efficiency would be to prepare the NCs in diverse ‘*solvents-of-synthesis*’ and to check their activities systematically. A similar practice is in place, even today, where the reaction conditions, precursors, or capping agents are optimized to improve catalytic activities of noble metal nanocrystal. However the ‘*solvent-of-synthesis*’ as an independent activity facilitator is never screened and has been overlooked so far. On the other hand, for organic catalytic reactions, solvents are routinely screened and optimized for superior yields. Through this study, the authors wish to propose that such screening of the ‘*solvent-of-synthesis*’, if undertaken in a routine manner while using noble metal nanocrystals, could be highly beneficial, leading to large improvements in activities.

## Conflicts of interest

There are no conflicts to declare.

## Supplementary Material

NA-003-D0NA01006E-s001
