# Supplementary material for: ‘Pre-optimization’ of the solvent of nanoparticle synthesis for superior catalytic efficiency: a case study with Pd nanocrystals
Source: Nanoscale Adv. 2021 Feb 17;3(8):2366–76. doi: 10.1039/d0na01006e (PMC9417607; doi:10.1039/d0na01006e)
Supplement: NA-003-D0NA01006E-s001 [file NA-003-D0NA01006E-s001.pdf]

Electronic Supplementary Material (ESI) for Nanoscale Advances.
This journal is © The Royal Society of Chemistry 2021

# Supplementary information

## 'Pre-optimization' of the solvent of nanoparticle-synthesis for superior catalytic efficiency: A case study with Pd nanocrystals

Lipipuspa Sahoo,[$] Parmeet Kaur Dhindsa,[$] Nihal C.P., Ujjal K. Gautam*

[$]These authors have contributed equally

Department of Chemical Sciences, Indian Institute of Science Education and Research (IISER)-Mohali, Sector 81, Mohali, SAS Nagar, Punjab 140306, India

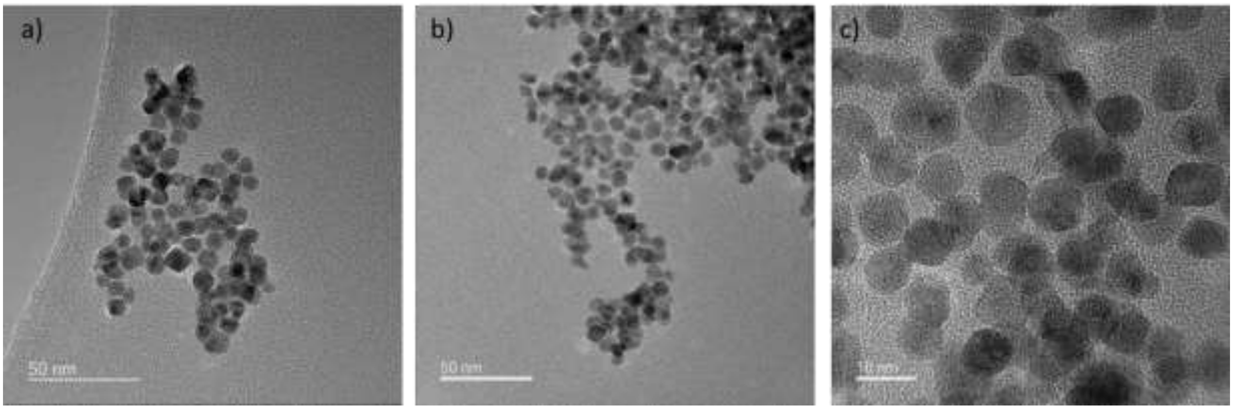


Fig. S1. TEM images of PdNC-1.

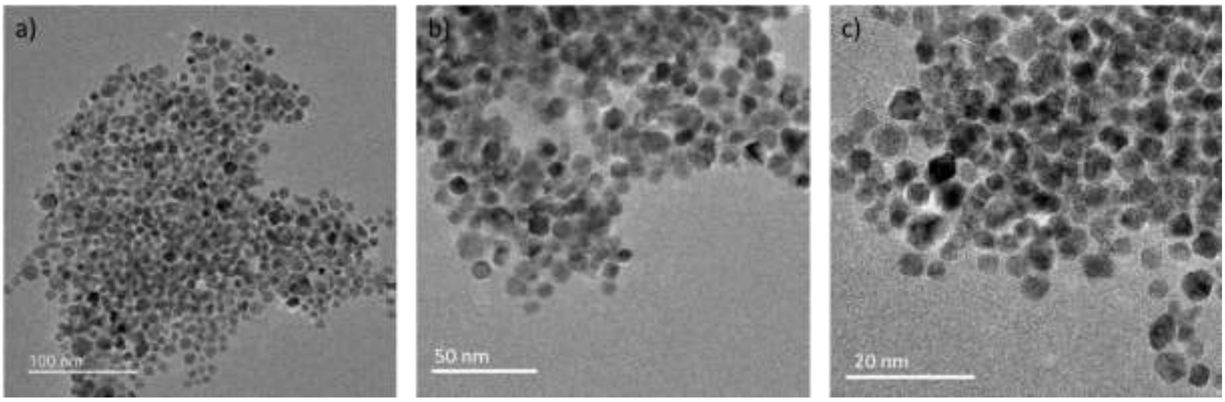


Fig. S2. TEM images of PdNC-2.

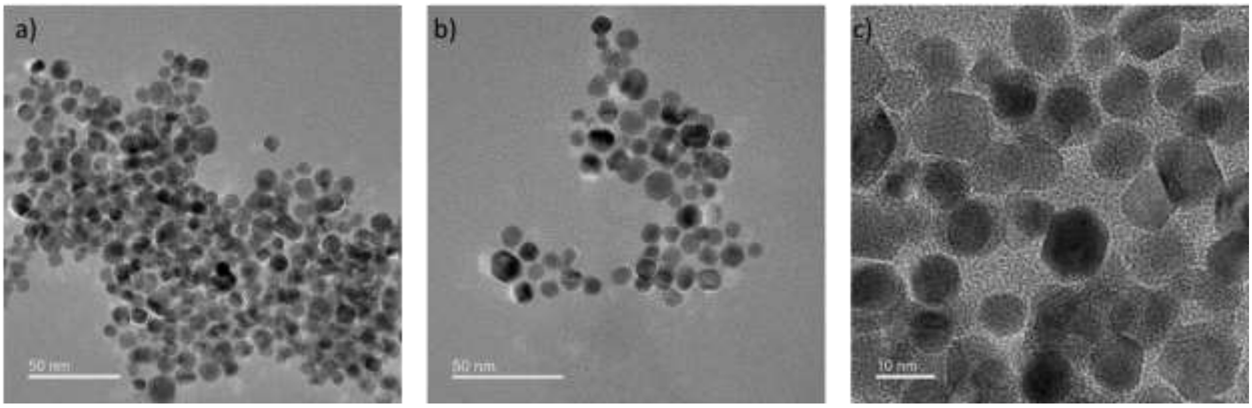


Fig. S3. TEM images of PdNC-3.

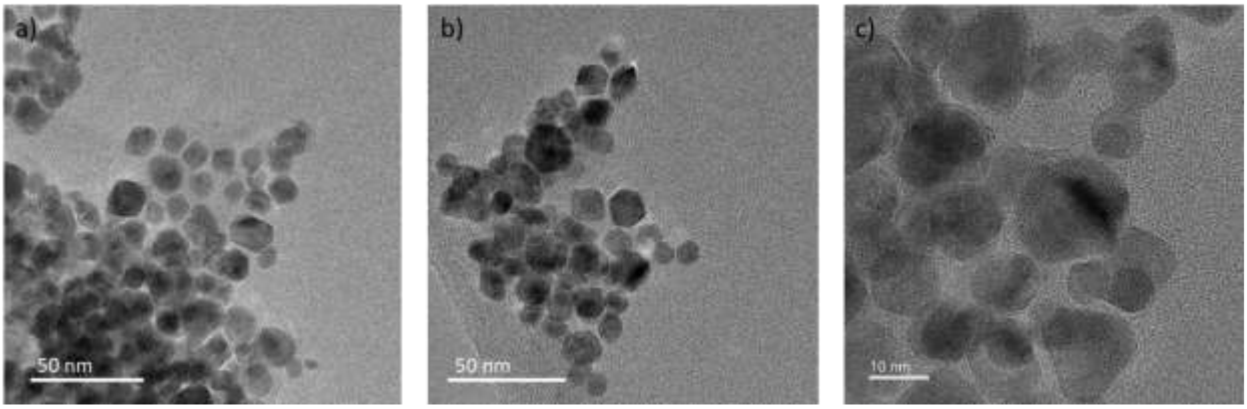


Fig. S4. TEM images of PdNC-4.

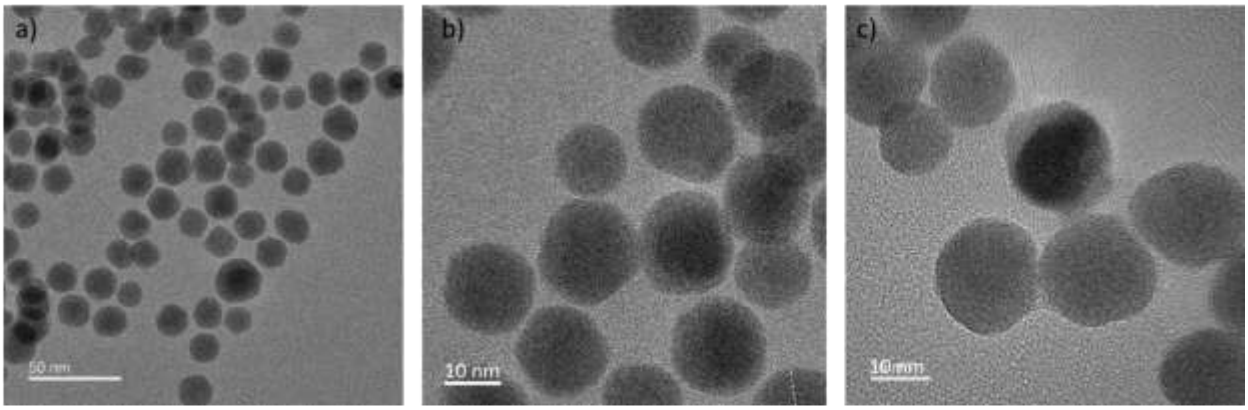


Fig. S5. TEM images of PdNC-5.

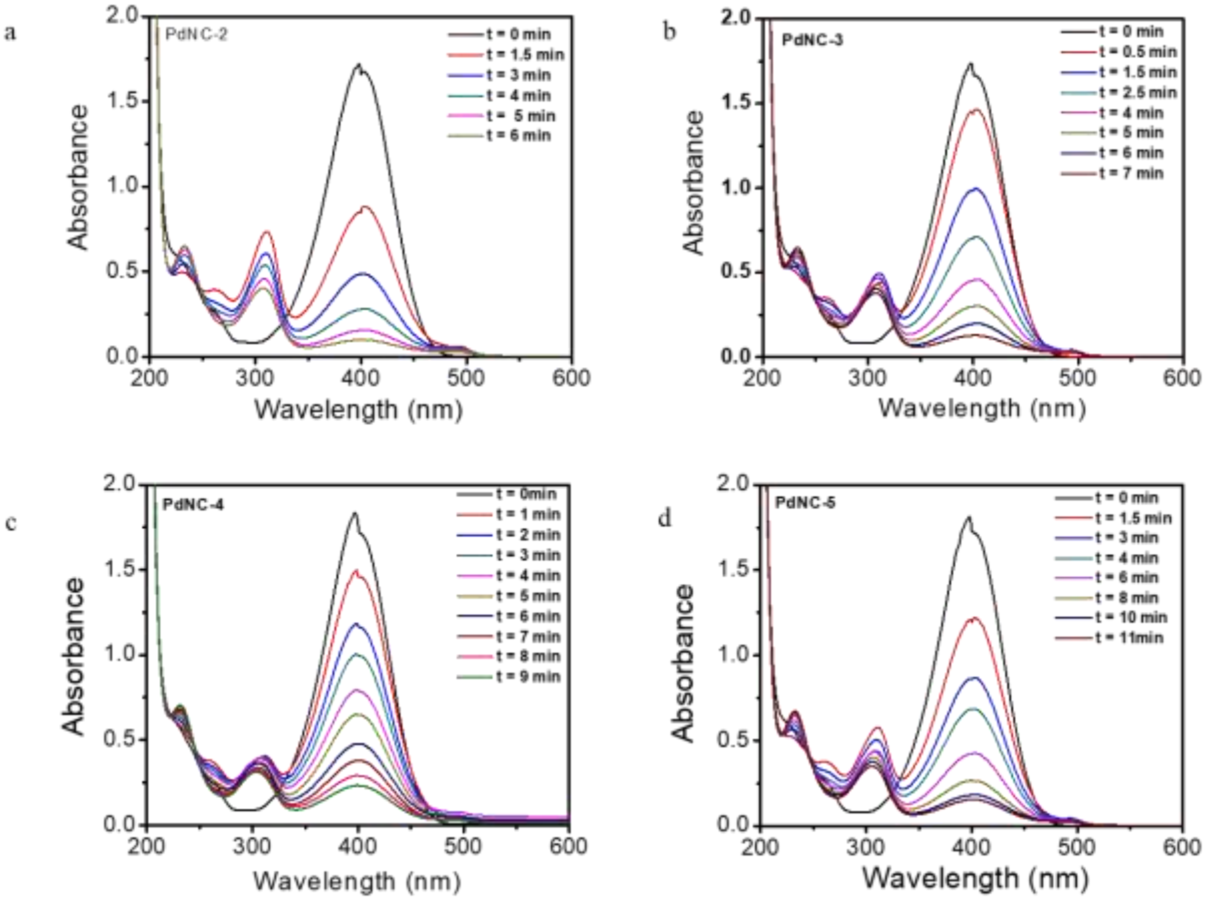


Fig. S6. Time dependent UV-Vis absorption spectra recorded during reduction of 4-NP by $NaBH_4$ in presence of (a) PdNC-2, (b) PdNC-3, (c) PdNC-4 and (d) PdNC-5.

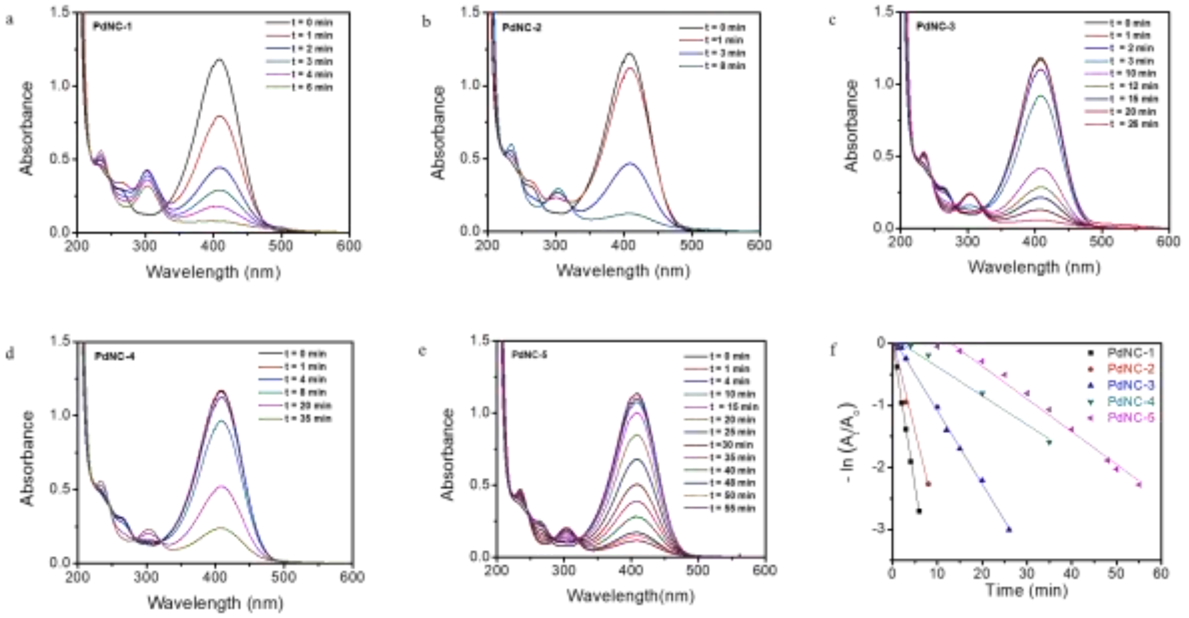


Fig. S7. Time dependent UV-Vis absorption spectra recorded during reduction of 2,5- NB by $NaBH_4$ in presence of (a) PdNC-1, (b) PdNC-2, (c) PdNC-3, (d) PdNC-4, (e) PdNC-5, and (f) the corresponding rate-constant plots of $-\ln(A_t/A_o)$ *vs.* time.

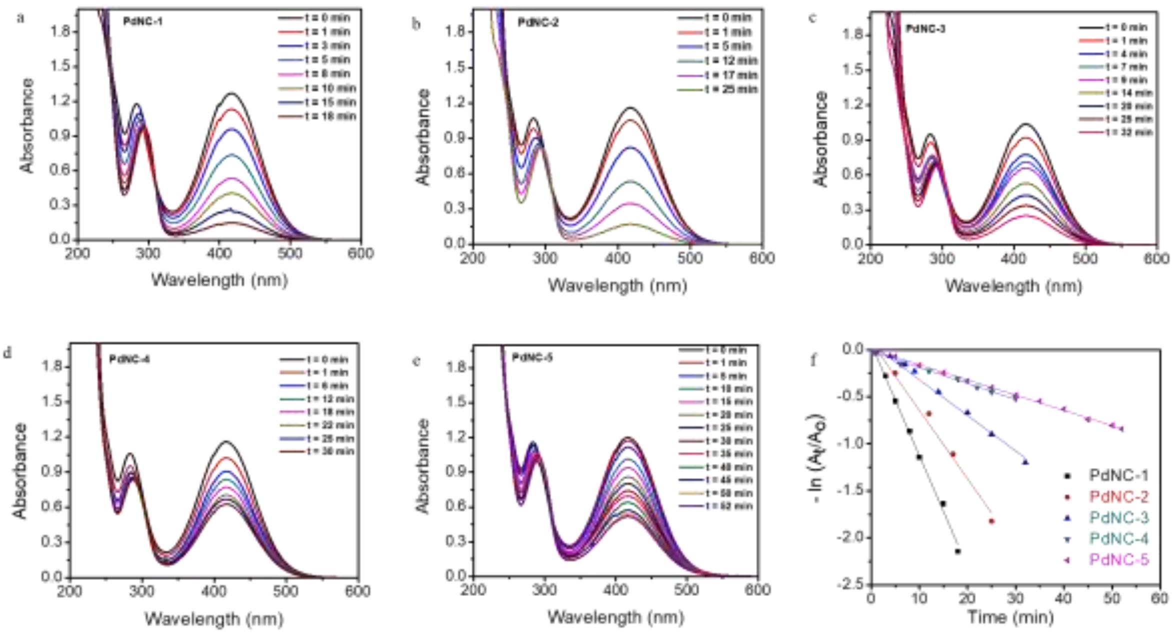


Fig. S8. Time dependent UV-Vis absorption spectra recorded during reduction of 2-NP by $NaBH_4$ in presence of (a) PdNC-1, (b) PdNC-2, (c) PdNC-3, (d) PdNC-4, (e) PdNC-5, and (f) the corresponding rate-constant plots of -ln($A_t$/$A_o$) *vs.* time.

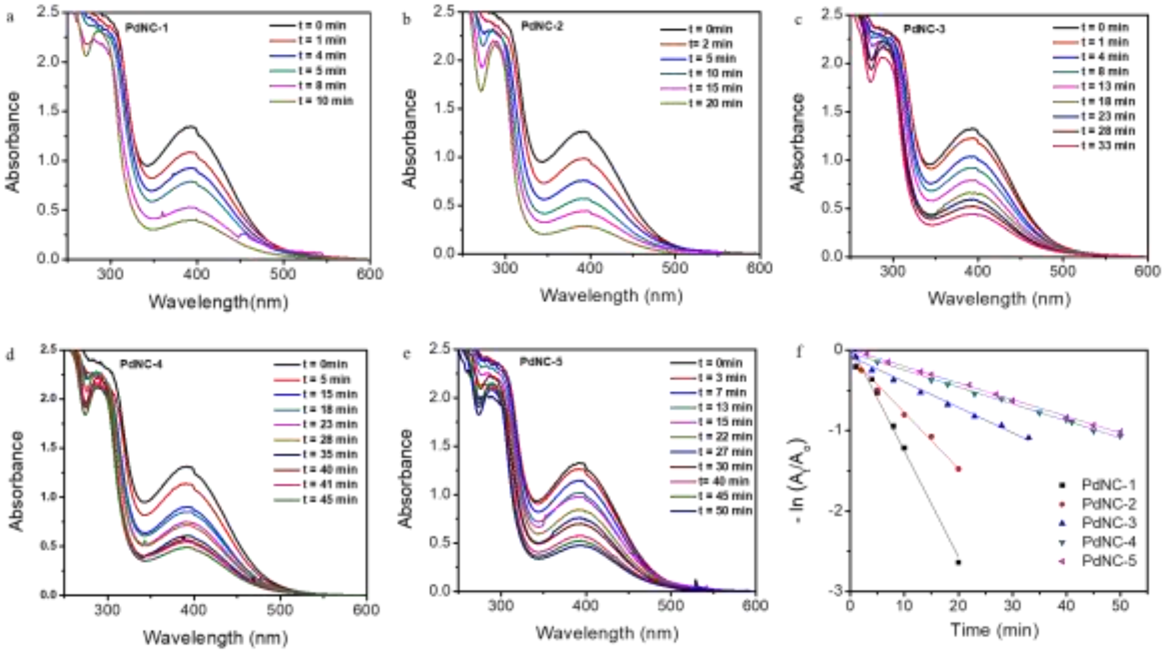


Fig. S9. Time dependent UV-Vis absorption spectra recorded during reduction of 3-NP by $NaBH_4$ in presence of (a) PdNC-1, (b) PdNC-2, (c) PdNC-3, (d) PdNC-4, (e) PdNC-5, and (f) the corresponding rate-constant plots of -ln ($A_t/A_o$) *vs*. time.

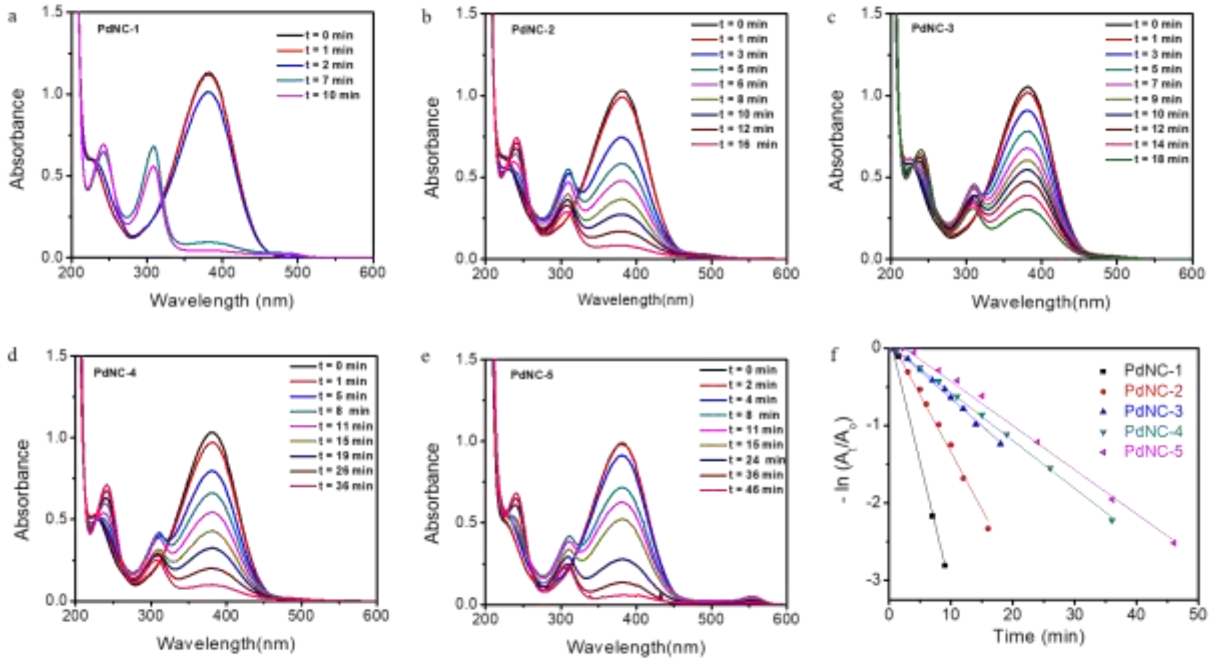


Fig. S10. Time dependent UV-Vis absorption spectra recorded during reduction of 4-NA by $NaBH_4$ in presence of (a) PdNC-1, (b) PdNC-2, (c) PdNC-3, (d) PdNC-4, (e) PdNC-5, and (f) the corresponding rate-constant plots of -ln($A_t$/$A_o$) *vs.* time.

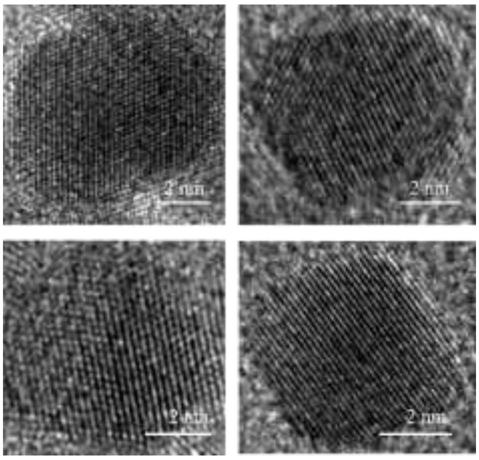


Fig. S11. Typical high-resolution TEM images of PdNC-1 on a few individual particles.

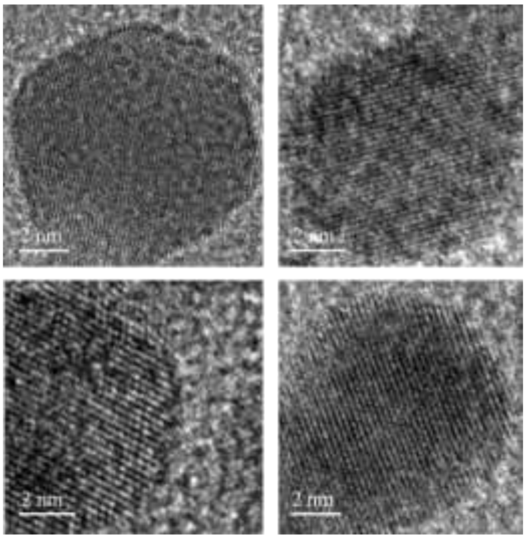


Fig. S12. Typical high-resolution TEM images of PdNC-2 on a few individual particles.

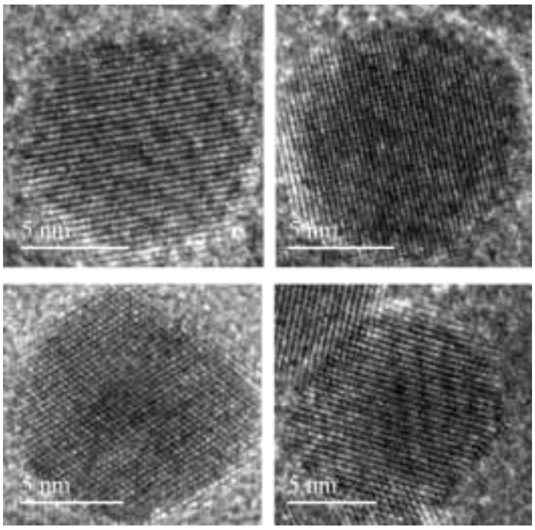


Fig. S13. Typical high-resolution TEM images of PdNC-3 on a few individual particles.

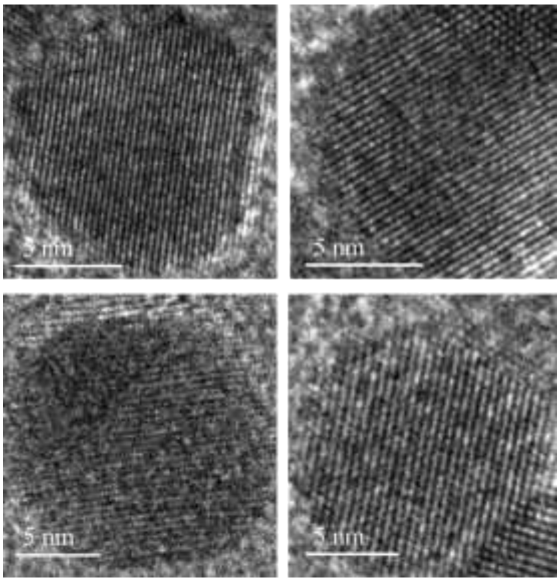


Fig. S14. Typical high-resolution TEM images of PdNC-4 on a few individual particles.

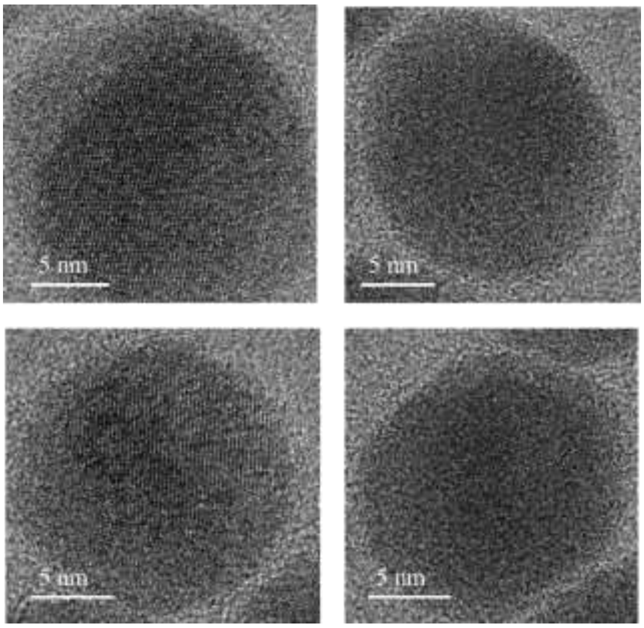


Fig. S15. Typical high-resolution TEM images of PdNC-5 on a few individual particles.

Supporting Note 1:

The efficiency of the catalysts were evaluated by calculating turnover frequency (TOF), where TOF can be defined as the moles of product formed per moles of catalyst atoms per second or hour as given below:

$$\mathrm{TOF} = \frac{moles\ of\ product}{moles\ of\ catalyst\ atoms \times time\ (sec\ (or\ hour))}$$

where moles of product is given by yield times the moles of substrate taken.

We further define surface turnover-frequency (s-TOF) as a number obtained by dividing TOF by fraction of surface atoms. It indicates the number of molecules reacted at each available catalytic site per unit time, assuming that the number of active catalytic sites is equal to total number of surface atoms.

$$\text{s-TOF} = \frac{\mathrm{TOF}}{\text{Fraction of surface atoms}}$$

For estimation of the number of surface atoms, an ideal spherical shape for the nanoparticles was considered as an approximation (note that since it is actually not true, it automatically factors in the deviation from an ideal shape induced by the solvents-of-synthesis). From the total number of atoms present within a spherical nanoparticle, the number of atoms below the surface layer of the sphere was subtracted (see schematic below). The number of atoms within a sphere was obtained by calculating the volume of the sphere from the radius using the volume formula, then multiplying the volume by the density of the metal to give the mass of the nanoparticle. The mass was converted to moles and finally to the number of atoms using Avogadro's number.

Average mass of a Pd NC

= Average volume of each particle × *d* (*d*=density of Pd)

$= [(x_1 V_1 + x_2 V_2 + x_3 V_3 \ldots\ldots..)/(x_1 + x_2 + x_3 \ldots\ldots..)] \times d$

($x_i$ = number of particles with volume $V_i$ ($=4/3\pi r_i^3$) from TEM analysis)

$$\text{Total number of atoms present in a Pd NC} = \frac{\text{Average mass of a Pd NC} \times N_a}{Atomic\ mass}$$

For estimating number of atoms present below the surface layer, we have defined subsurface radius as the nanoparticle radius minus one diameter of a Pd atom. The covalent radius of Pd atom is 0.138 nm, so a nanoparticle with radius ‘R’ would have a subsurface radius, $r_{subsurface} = R - 2 \times 0.138$ nm (see the schematic given below). Number of subsurface atoms are calculated as above, but using the subsurface radius ‘$r_{subsurface}$’ instead of radius ‘r’ in the average volume calculation. The number of surface atoms is the difference between the total number of atoms and number of subsurface atoms. The fraction of surface atoms were calculated as given below.

$$\text{Fraction of surface atoms} = \frac{Total\ number\ of\ surface\ atoms}{Total\ number\ of\ atoms}$$

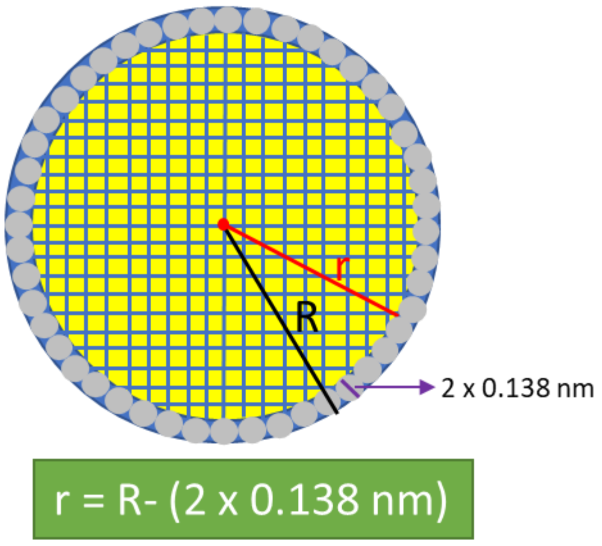

Table S1. Apparent rate-constant values exhibited by the different PdNCs for nitroarene reduction reaction using $NaBH_4$.

| Sample | Apparent Rate constant ($min^{-1}$) | | | | |
|---|---|---|---|---|---|
| | 4-NP | 2,5-NB | 2-NP | 3-NP | 4-NA |
| PdNC-1 | 0.71 | 0.46 | 0.12 | 0.13 | 0.33 |
| PdNC-2 | 0.46 | 0.29 | 0.07 | 0.07 | 0.15 |
| PdNC-3 | 0.30 | 0.12 | 0.04 | 0.03 | 0.07 |
| PdNC-4 | 0.21 | 0.05 | 0.02 | 0.02 | 0.06 |
| PdNC-5 | 0.20 | 0.04 | 0.02 | 0.02 | 0.06 |

Table S2. Fraction of surface atoms present in all PdNCs.

| Sample | Average diameter (nm) | No. of atoms per NCs | No. of surface atoms per NCs | Fraction of surface atoms |
|---|---|---|---|---|
| PdNC-1 | 6.4 ± 1.0 | $9.16 \times 10^3$ | $2.18\times 10^3$ | 0.243 |
| PdNC-2 | 7.9 ± 1.6 | $1.73 \times 10^4$ | $3.39\times 10^3$ | 0.203 |
| PdNC-3 | 9.8 ± 2.1 | $3.34 \times 10^4$ | $5.34\times 10^3$ | 0.166 |
| PdNC-4 | 12.4 ± 2.6 | $6.53 \times 10^4$ | $8.84\times 10^3$ | 0.134 |
| PdNC-5 | 16.9 ± 2.2 | $1.73 \times 10^5$ | $1.64 \times 10^4$ | 0.096 |

Table S3. s-TOF and relative s-TOF (with respect to PdNC-1) for Suzuki-Miyaura coupling reaction using the different PdNCs.

| Sample | s-TOF ($h^{-1}$) | Relative s-TOF |
|---|---|---|
| PdNC-1 | 997 | 1 |
| PdNC-2 | 630 | 0.63 |
| PdNC-3 | 403 | 0.40 |
| PdNC-4 | 248 | 0.25 |
| PdNC-5 | 94 | 0.09 |

Table S4. TOF exhibited by the diferent PdNCs for nitroarene reduction reaction using $NaBH_4$.

| Sample | 4-NP TOF ($min^{-1}$) | 2,5-NB TOF ($min^{-1}$) | 2-NP TOF ($min^{-1}$) | 3-NP TOF ($min^{-1}$) | 4-NA TOF ($min^{-1}$) |
|---|---|---|---|---|---|
| PdNC-1 | 2.15 | 1.29 | 1.89 | 1.99 | 1.97 |
| PdNC-2 | 1.29 | 0.80 | 1.08 | 1.08 | 0.92 |
| PdNC-3 | 0.80 | 0.34 | 0.61 | 0.46 | 0.41 |
| PdNC-4 | 0.58 | 0.14 | 0.31 | 0.31 | 0.36 |
| PdNC-5 | 0.53 | 0.11 | 0.31 | 0.31 | 0.36 |

Table S5. s-TOF exhibited by the different PdNCs for nitroarene reduction reaction using $NaBH_4$.

| Sample | 4-NP s-TOF ($min^{-1}$) | 2,5-NB s-TOF ($min^{-1}$) | 2-NP s-TOF ($min^{-1}$) | 3-NP s-TOF ($min^{-1}$) | 4-NA s-TOF ($min^{-1}$) |
|---|---|---|---|---|---|
| PdNC-1 | 8.86 | 5.31 | 7.77 | 8.20 | 8.13 |
| PdNC-2 | 6.36 | 3.97 | 5.35 | 5.35 | 4.54 |
| PdNC-3 | 4.85 | 2.04 | 3.72 | 2.80 | 2.52 |
| PdNC-4 | 4.37 | 1.04 | 2.32 | 2.32 | 2.71 |
| PdNC-5 | 5.59 | 1.15 | 3.24 | 3.24 | 3.78 |

Table S6. Relative s-TOF values exhibited by the different PdNCs for nitroarene reduction reaction using $NaBH_4$.

| Sample | 4-NP s-TOF | 2,5-NB s-TOF | 2-NP s-TOF | 3-NP s-TOF | 4-NA s-TOF |
|---|---|---|---|---|---|
| PdNC-1 | 1 | 1 | 1 | 1 | 1 |
| PdNC-2 | 0.72 | 0.75 | 0.69 | 0.66 | 0.56 |
| PdNC-3 | 0.55 | 0.39 | 0.48 | 0.34 | 0.32 |
| PdNC-4 | 0.49 | 0.19 | 0.30 | 0.28 | 0.34 |
| PdNC-5 | 0.62 | 0.21 | 0.41 | 0.39 | 0.46 |
